# Steeper medial posterior tibial slope is associated with medial meniscal extrusion and early‐onset knee osteoarthritis in middle‐aged Japanese women: A longitudinal cohort study

**DOI:** 10.1002/jeo2.70772

**Published:** 2026-05-19

**Authors:** Hikaru Kristi Ishibashi, Kyohei Ishibashi, Kyota Ishibashi, Yukiko Sakamoto, Eiji Sasaki, Yuka Kimura, Gentaro Kumagai, Eiichi Tsuda, Mizuri Ishida, Tsukasa Tanaka, Yasuyuki Ishibashi

**Affiliations:** ^1^ Department of Orthopaedic Surgery Hirosaki University Graduate School of Medicine Hirosaki Aomori Japan; ^2^ Department of Rehabilitation Medicine Hirosaki University Graduate School of Medicine Hirosaki Aomori Japan; ^3^ Research Institute of Health Innovation Hirosaki University Hirosaki Aomori Japan; ^4^ Innovation Center for Health Promotion Hirosaki University Graduate School of Medicine Hirosaki Aomori Japan

**Keywords:** medial meniscus extrusion, osteoarthritis, posterior tibial slope, tibial morphology, ultrasonography

## Abstract

**Purpose:**

This study primarily aimed to evaluate the longitudinal association between proximal tibial morphologic parameters and medial meniscal extrusion for 5 years and examine the relationship of both proximal tibial morphologic parameters and medial meniscal extrusion with the onset of knee osteoarthritis (OA) in middle‐aged Japanese women. The secondary aim was to identify factors associated with changes in medial meniscal extrusion.

**Methods:**

A longitudinal retrospective cohort of middle‐aged Japanese women without radiographic OA at baseline was followed for 5 years. No radiographic OA was defined as a Kellgren–Lawrence grade of 0 or 1 on plain radiographs. Proximal tibial morphologic parameters, including the medial and lateral posterior tibial slope (PTS) and medial proximal tibial angle (MPTA), were assessed using magnetic resonance imaging. OA onset was defined as progression to Kellgren–Lawrence grade ≥2 on follow‐up plain radiographs. Medial meniscal extrusion was measured using ultrasonography at baseline and at 5 years, and the change in medial meniscal extrusion was calculated. Linear regression analyses were performed to identify independent predictors and optimal thresholds for OA onset. A *p* value < 0.05 was considered statistically significant.

**Results:**

In total, 134 women (mean age 54.1 ± 8.9 years; mean body mass index 21.6 ± 2.8 kg/m^2^) were included. A steeper medial PTS (mPTS) was associated with greater change in medial meniscal extrusion (*p* < 0.01), whereas MPTA (*p* = 0.73) and lateral PTS (*p* = 0.20) were not associated with changes in medial meniscus extrusion. The mPTS and change in medial meniscal extrusion were independent predictors of OA onset (both *p* < 0.01).

**Conclusions:**

Steeper mPTS and greater change in medial meniscal extrusion may be critical structural risk factors for the early development of knee OA in middle‐aged Japanese women. Incorporating assessments of mPTS and change in medial meniscal extrusion into clinical protocols may aid in the early initiation of preventive interventions for knee OA.

**Level of Evidence:**

Level IV.

## Graphical abstract

The change in MME was associated with steeper mPTS and was also associated with the onset of knee OA.

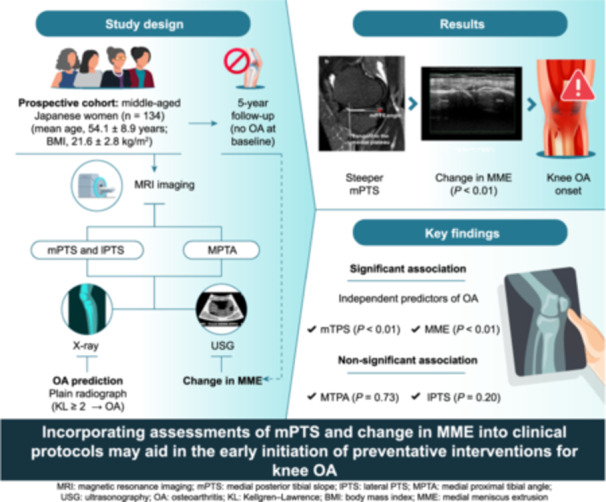

**Abbreviations**
lPTS: lateral posterior tibial slope
MME: medial meniscal extrusion
MPTA: medial proximal tibial angle
mPTS: medial posterior tibial slope
MRI: magnetic resonance imaging
OA: osteoarthritis
PTS: posterior tibial slope
ROC: receiver operating characteristic

## INTRODUCTION

Osteoarthritis (OA) is a degenerative joint disease characterized by progressive cartilage loss, subchondral bone remodelling, and impaired joint function [17]. Female sex [33, 34] and increasing age [14] are well‐established risk factors, and a high OA burden among middle‐aged and older women highlights the need to identify early structural predictors of knee OA to enable prevention and targeted intervention.

Lower limb alignment plays a critical role in the pathogenesis of knee OA [7, 25], and proximal tibial morphology has emerged as an important structural determinant. A decreased medial proximal tibial angle (MPTA), reflecting varus alignment, is associated with medial joint space narrowing and OA progression [28]. In the sagittal plane, a steeper posterior tibial slope (PTS) increases anterior tibial translation and shear stress on the medial meniscus [13, 26]. Medial PTS (mPTS) has also been linked to bone marrow lesions in middle‐aged women with early‐stage knee OA [18], suggesting a biomechanical contribution of tibial morphology to disease development.

Medial meniscus extrusion (MME) is another key structural abnormality associated with knee OA. Meniscus extrusion impairs load distribution and promotes cartilage degeneration [9, 10, 11]. Although magnetic resonance imaging (MRI) is commonly used to assess MME, ultrasonography provides a non‐invasive and practical alternative for large‐scale population studies. Baseline MME measured by ultrasonography has been associated with future knee OA onset [5, 6], and in overweight and obese women, baseline MME predicts disease development [36].

Furthermore, among overweight and obese women, baseline MME has been shown to predict knee OA development [36], highlighting the relevance of MME as a risk factor in susceptible female populations. Despite growing evidence linking MME to knee OA, an important gap remains. Although baseline MME has been associated with knee OA incidence, longitudinal changes in MME and their relationship to knee OA onset have not been adequately explored. Moreover, the interaction between changes in MME and proximal tibial morphology (PTS and MPTA) has not yet been investigated as a combined predictive factor for future knee OA development.

Accordingly, the present study aimed to investigate (1) the association between tibial morphology (PTS and MPTA) and longitudinal changes in MME in a community‐dwelling cohort of middle‐aged women and (2) the relationship between the magnitude of MME change over a 5‐year period and the incidence of knee OA. This study hypothesized that smaller MPTA and steeper PTS at baseline would be associated with changes in MME and that the degree of MME change would predict the future onset of knee OA.

## METHODS

### Study design and participants

This longitudinal retrospective cohort study was conducted as part of the Iwaki Health Promotion Project. This annual community‐based medical examination began in 2005 and involves more than 1000 volunteers each year [19, 31]. Participants included middle‐aged individuals without radiographic knee OA in 2017 (defined as baseline) who were successfully followed up in 2022 (defined as the 5‐year follow‐up). The Kellgren–Lawrence (KL) grading scale was used to diagnose knee OA, and KL grades 0 or 1 were defined as no radiographic knee OA [22]. The exclusion criteria were as follows: male sex, lack of MRI data, lack of radiographic data, history of knee injury, rheumatoid arthritis or radiographic knee OA with a KL grade ≥ 2. Twenty‐one cases showed degenerative meniscal lesions without evidence of meniscal tears, and all were included in the analysis. Ethical approval for this study was granted by the ethics committee of Hirosaki University (reference numbers: 2021‐030 and 2021‐166‐3). All procedures were conducted in accordance with the Declaration of Helsinki (1964) and its later revisions. Written informed consent was obtained from all participants prior to study inclusion.

### Radiographic evaluations

Plain radiographs of the right knee were obtained at baseline (2017) and at the 5‐year follow‐up (2022). Imaging was performed with full knee extension under weight‐bearing conditions, with standardized foot map positioning, using a mobile radiographic unit (CXDI‐40EG digital radiography system; Canon Inc.). Imaging parameters were set at 60 kV, 50 mA and 80 ms. Two experienced orthopaedic surgeons (H.K.I. and E.S.) independently assessed all radiographs, and participants with a baseline KL grade ≥2 were excluded. A KL grade ≥2 on plain radiographs at the 5‐year follow‐up was considered indicative of knee OA onset.

### MRI measurements of proximal tibial morphology

All participants underwent MRI of the right knee within 1 week of the initial examination. Imaging was performed using a mobile MR unit (1.5 T; ECHELON RX, Hitachi) equipped with a rapid extremity coil. Participants were positioned supine with the knee in full extension. The imaging protocol included sagittal and coronal T2‐weighted fat‐saturated fast spin echo sequences (repetition time, 5000 ms; echo time, 80 ms; field of view, 16 cm; matrix, 288 × 288; slice thickness, 3 mm; interslice gap, 1.0 mm).

**Figure 1:**
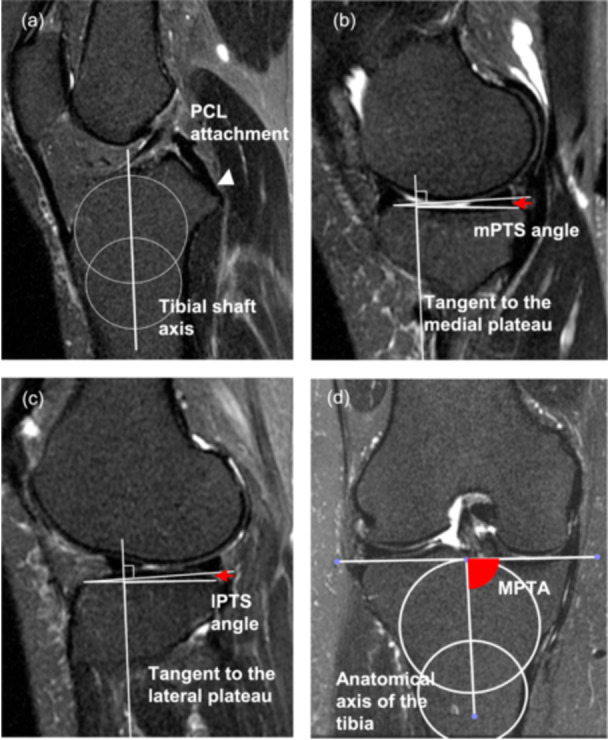
Measurements of proximal tibial morphological parameters performed using MRI. The central sagittal plane is shown on the slice corresponding to the attachment site of the posterior cruciate ligament and the intercondylar eminence. On this slice, two circles are fitted to the anterior and posterior cortices of the tibia, and the line connecting the centres of these circles is defined as the tibial axis (a). The medial and lateral posterior tibial slopes (mPTS and lPTS) are calculated as the angles between a line perpendicular to the tibial axis and the line connecting the most proximal anterior and posterior subchondral bone points at the centres of the medial and lateral tibiofemoral compartments, respectively (b and c). The medial proximal tibial angle (MPTA) is defined as the angle formed between the anatomical axis of the tibia and the proximal tibial joint line on the coronal MRI view (d). MRI, magnetic resonance imaging.

The mPTS and lateral PTS (lPTS) were measured using a previously established method [16, 24]. Briefly, the central sagittal plane was identified on MRI slices based on anatomical landmarks, including the posterior cruciate ligament attachment and the intercondylar eminence (Figure 1a). Within this plane, two best‐fit circles were placed along the anterior and posterior cortices of the proximal tibia, and a line connecting the centres of these circles was defined as the tibial shaft axis. The mPTS and lPTS were then calculated as the angle between a line perpendicular to the tibial axis and a line connecting the most proximal anterior and posterior points of the subchondral bone at the centre of the medial and lateral tibiofemoral compartments, respectively (Figure 1b,c).

The MPTA was measured on coronal slices showing the medial and lateral menisci (Figure 1d). The MPTA was defined as the angle between the tibial anatomic axis and the joint line of the tibial plateau [1]. To ensure accurate identification of the tibial longitudinal axis, MRI acquisition encompassed the proximal one third of the tibia. All images met predefined quality criteria, and no knees were excluded because of suboptimal image quality.

Two independent observers (H.K.I., 5 years of experience; K.I., 1 year of experience) assessed inter‐rater reliability using 40 randomly selected images. Measurements were repeated at two separate sessions with a 1‐week interval. The examiner was blinded to the initial measurements at the time of the second assessment. Inter‐rater reliability for the MRI measurements was excellent, with an intraclass correlation coefficient (ICC [2, 1]) of 0.92 (95% confidence interval [CI] = 0.88–0.99). The ICCs for HKI. (1, 1) and KI (1, 1) were 0.93 (95% CI = 0.85–0.99) and 0.93 (95% CI = 0.89–0.95), respectively [18].

### Ultrasonographic measurements of the MME

Medial meniscus extrusion was measured using ultrasonography as a non‐invasive and reliable imaging technique, as previously described [5, 21]. All knees were evaluated at baseline (2017) and follow‐up (2022) by two independent examiners (K.I., 6 years of experience; H.K.I., 3 years of experience at baseline) using a linear transducer (frequency, 7–12 MHz; Viamo™, Canon Medical Systems Corp.). Participants were positioned supine with both knees fully extended and the feet resting in a naturally externally rotated position. The probe was placed perpendicular to the joint line over the medial joint space to clearly visualize the medial collateral ligament. On the captured images, a reference line was drawn connecting the femoral and tibial cortices. MME was defined as the length of a perpendicular line drawn from this reference line to the outermost edge of the medial meniscus. All measurements were performed independently by two examiners blinded to other study results to ensure reliability. Longitudinal change in MME (ΔMME) between 2017 and 2022 was calculated. Inter‐rater reliability for MME was good, with an ICC (2,1) of 0.86 (95% CI = 0.67–0.94). The ICCs for HKI (1, 1) and KI (1, 1) were 0.98 (95% CI = 0.94–0.99) and 0.98 (95% CI = 0.95–0.99), respectively [5]. To estimate ICCs, two examiners measured MME values in 18 participants on 2 separate days, 1 week apart. Measurements from the 1st day were blinded during the second assessment. In this subset, the standard deviation of medial meniscal extrusion was 0.55 mm. Based on these data, the standard error of measurement for ultrasonographic MME was 0.20 mm. The minimal detectable change was 0.47 mm at the 90% confidence level and 0.56 mm at the 95% confidence level.

### Statistical analysis

All data were shown as mean ± standard deviation. The association between ΔMME and proximal tibial morphologic parameters was assessed using Spearman's rank correlation analysis. The Spearman rank correlation analysis was used for continuous variables, because some demographic parameters were not normally distributed according to the Shapiro–Wilk test.

Multivariable linear regression analysis was performed to identify independent predictors of ΔMME after adjustment for potential confounders, including age and body mass index (BMI). To evaluate factors associated with knee OA during follow‐up, regression analysis was performed using knee OA onset as the dependent variable and ΔMME and proximal tibial morphologic parameters as explanatory variables. An a priori sample size estimation for multiple linear regression with seven predictors indicated that 103 participants would be required to detect a medium effect size (Cohen's *f* ^2^ = 0.15) at an alpha level of 0.05 with 80% power. The final sample size of 134 participants exceeded this requirement.

Statistical analyses were performed using SPSS version 25.0 (IBM Corp.). A *p* value < 0.05 was considered statistically significant.

## RESULTS

**Figure 2:**
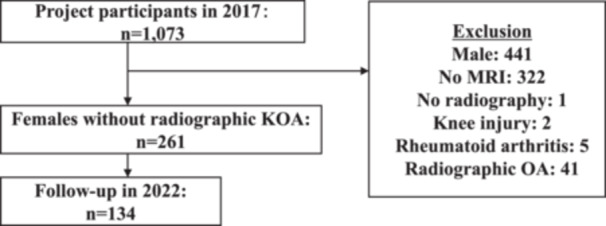
Flowchart of the present study. MRI, magnetic resonance imaging; OA, osteoarthritis.

Of the 1073 participants without radiographic knee OA in 2017, 812 were excluded because of male sex (*n* = 441), lack of MRI data (*n* = 322), lack of radiographic data (*n* = 1), history of knee injury (*n* = 2), rheumatoid arthritis (*n* = 5) or radiographic knee OA with a KL grade ≥2 (*n* = 41). Overall, 261 women had adequate baseline data; among these, 134 were successfully followed up in 2022 and were included in the final analysis (Figure 2). At baseline, the mean age of the participants was 54.1 ± 8.9 years, and the mean BMI was 21.6 ± 2.8 kg/m^2^.

At baseline, proximal tibial morphology measurements obtained using MRI were listed in Table 1. MME was 3.4 ± 1.1 mm in 2022, resulting in a ΔMME of 0.6 ± 1.1 mm. The mean KL grade was 0.4 ± 0.5 at baseline and 1.3 ± 0.8 at the 5‐year follow‐up.

**Table 1:** Demographic data of middle‐aged Japanese women at baseline.

Age (y)	54.1 ± 8.9
Height (cm)	157.4 ± 5.0
BMI (m^2^/kg)	21.6 ± 2.8
MPTA (°)	86.3 ± 1.3
mPTS (°)	3.4 ± 3.5
lPTS (°)	1.8 ± 3.2
MME in 2017 (mm)	2.7 ± 0.7

Abbreviations: BMI, body mass index; cm, centimetre; lPTS, lateral posterior tibial slope; mm, millimetre; m^2^/kg, kilograms per square metre; MME, medial meniscus extrusion; MPTA, medial proximal tibial angle; mPTS, medial posterior tibial slope; y, years; ΔMME, change in medial meniscus extrusion.

Correlation analysis revealed a significant association between mPTS and ΔMME, as indicated by Spearman rank correlation coefficients (*ρ*) summarized in Table 2. mPTS showed a statistically significant correlation with ΔMME (*ρ* = 0.25, *p* = 0.02). In contrast, MPTA (*ρ* = 0.03, *p* = 0.73) and lPTS (*ρ* = 0.11, *p* = 0.20) showed no significant correlation.

**Table 2:** Correlation between the proximal tibial morphologic parameters and ΔMME.

	*ρ*	*p*
MPTA	−0.03	0.73
mPTS	0.25	<0.01*
lPTS	0.11	0.20

Abbreviations: lPTS, lateral posterior tibial slope; MPTA, medial proximal tibial angle; mPTS, medial posterior tibial slope; ρ, Spearman's rank correlation coefficient; ΔMME, change in medial meniscus extrusion.

* Statistically significant (<0.05).

Linear regression analysis, adjusting for confounders such as BMI and age, identified mPTS (*β* = 0.30, *p* < 0.01) as an independent predictor of ΔMME and ΔMME (*β* = 0.28, *p* < 0.01) as an independent predictor of knee OA development (Table 3). The regression model demonstrated a coefficient of determination (*R* ^2^) of 0.176, corresponding to a Cohen's *f* ^2^ of 0.21, indicating a moderate effect size.

**Table 3:** Linear regression analysis of the relationship between proximal tibial morphologic parameters and ΔMME.

	*β*	*p*	95% CI
Age	−0.07	0.63	−0.04 to 0.03
BMI	0.82	0.54	−0.68 to 1.29
BMD	0.06	0.65	−2.54 to 4.09
MPTA	−0.03	0.76	−0.17 to 0.12
mPTS	0.30	<0.01*	0.03–0.16
lPTS	−0.05	0.65	−0.09 to 0.06
OA onset	0.28	<0.01*	0.15–1.06

Abbreviations: BMD, bone mineral density; BMI, body mass index; CI, confidence interval; lPTS, lateral posterior tibial slope; MPTA, medial proximal tibial angle; mPTS, medial posterior tibial slope; OA, osteoarthritis; ΔMME, change in medial meniscus extrusion.

* Statistically significant (<0.05).

## DISCUSSION

This longitudinal cohort study revealed a significant association between steeper mPTS and greater ΔMME over a 5‐year period in middle‐aged Japanese women without radiographic knee OA at baseline and identified ΔMME as an independent predictor of OA onset. In contrast, MPTA and lPTS were not significantly associated with ΔMME. Although the 5‐year ΔMME was small (0.6 ± 1.1 mm), the measurement precision analysis indicated that ultrasonography can detect longitudinal changes in medial meniscal extrusion beyond measurement error.

These findings suggest that ΔMME may serve as an important predictor of the development and progression of knee OA. Previous studies have reported that one of the earliest changes in knee OA—namely joint space narrowing—may be attributable to medial meniscal extrusion rather than cartilage thinning [1, 8]. Shimozaki et al. found that >75% of patients with early‐stage knee OA exhibited ≥2 mm of MME [32], and Chiba et al. reported that a baseline MME of ≥4 mm conferred an odds ratio of 6.8 for OA onset after 5 years [6]. In the present study, a greater ΔMME was associated with the development of knee OA, underscoring the importance of longitudinal MME assessment to facilitate early intervention, including patient education and other preventive strategies aimed at reducing the risk of knee OA onset.

With respect to knee alignment, only mPTS was associated with ΔMME, whereas no associations were observed for MPTA or lPTS. The association between a steeper mPTS and increased ΔMME supports the biomechanical hypothesis that a steeper mPTS increases anterior shear forces on the medial meniscus, thereby promoting extrusion and destabilizing the medial compartment [13]. Kanayama et al. reported that individuals with medial meniscal degeneration exhibited a steeper mPTS and greater MME than those without degeneration [20]. In addition, several studies have demonstrated associations between a steeper mPTS and medial meniscus posterior root tears [26, 38]. Together, these findings suggest that a steeper mPTS leads to greater shear forces acting on the medial meniscus, which may promote meniscal extrusion as well as root tears. Although lPTS was not associated in the present analysis, relevant associations might have been identified had lateral meniscal evaluation been included. MPTA has been implicated in varus malalignment and increased medial compartment and meniscal loading [37]. Hiranaka et al. demonstrated that patients with knee OA had smaller MPTA values compared with individuals without knee OA [15]. However, because the current study included only participants without OA at baseline, alignment may have been more homogeneous, limiting variability.

In this study, MME was assessed using ultrasonography, with longitudinal follow‐up conducted over 5 years. Several previous investigations have demonstrated associations between MME and knee OA progression using MRI [4, 29]. However, ultrasonography offers advantages in portability, cost‐effectiveness, non‐invasiveness, and rapid execution, making it well‐suited for routine clinical use and longitudinal monitoring [12, 30]. Although MRI allows detailed structural assessment, its cost and limited accessibility restrict broader clinical application. Based on our findings, ΔMME appears to be a useful non‐invasive longitudinal biomarker for early detection of OA risk. In clinical practice, detection of an increase in ΔMME could prompt a subsequent MRI to further evaluate bone morphology, cartilage loss, and meniscal pathology.

This study focused exclusively on middle‐aged women to minimize potential confounding, as previous studies have reported sex‐based differences in bone morphology, with men exhibiting larger MPTA values [3, 15] and women demonstrating steeper PTS [9]. Age‐related changes in bone morphology have also been described [35], and middle‐aged women were selected because of their elevated risk of OA development.

Several limitations should be acknowledged. First, generalizability is limited because the study included only middle‐aged Japanese women. Owing to the cost and logistical constraints of MRI and ultrasonography, we targeted a high‐risk population for OA, which limits applicability to large‐scale epidemiological studies. Second, although approximately 1,000 individuals were enroled at baseline, only approximately half completed the 5‐year follow‐up. This attrition was related to the voluntary nature of participation, and although participation rates did not substantially differ by year, the study population at each time point was not identical. Third, MME was evaluated only at the central portion of the medial meniscus, where it attaches to the medial collateral ligament, in the supine position. Therefore, this study could not discuss how MME may change under weight‐bearing conditions or at other measurement sites. Finally, although lower limb alignment is widely recognized as a key factor in OA progression [25, 28], full‐length weight‐bearing radiographs were not obtained. Additionally, lateral knee radiography and follow‐up MRI at 5 years could not be performed due to the constraints of a large‐scale community screening programme, including limitations on cost, time, and radiation exposure.

Future research should include more diverse populations, a comprehensive evaluation of both medial and lateral meniscal extrusion, and integration of ΔMME and mPTS assessments into OA screening protocols.

Overall, this study suggests that mPTS may influence medial meniscal stability and play a key role in the pathogenesis of knee OA. Early identification and monitoring of individuals with a steep mPTS and increasing ΔMME may enable timely preventive interventions, such as optimal weight management [23] and lifestyle modifications involving diet and exercise [2, 27], potentially delaying OA progression.

## CONCLUSIONS

This study identified steeper mPTS as a significant structural risk factor for MME and early onset of knee OA in middle‐aged Japanese women. Furthermore, ΔMME measured using ultrasonography was an independent predictor of OA onset. These findings highlight the clinical value of incorporating mPTS and ΔMME assessment into OA risk evaluation protocols to enable early identification of individuals at increased risk for knee OA.

## AUTHOR CONTRIBUTIONS

Hikaru Kristi Ishibashi and Kyohei Ishibashi designed the study, supervised the data collection, and drafted the manuscript. Yukiko Sakamoto and Eiichi Tsuda participated in data collection, assisted with the statistical analysis and drafting of the manuscript, and critically revised it. Eiji Sasaki, Yuka Kimura and Gentaro Kumagai participated in data collection. Mizuri Ishida, Tsukasa Tanaka and Yasuyuki Ishibashi conceived and designed the study, supervised the team, facilitated the acquisition of data, and provided directions for the critical review of the manuscript. All the authors have read and approved the final version of the manuscript.

## CONFLICT OF INTEREST STATEMENT

The authors declare no conflicts of interest.

## ETHICS STATEMENT

All procedures involving human participants performed in this study were in accordance with the ethical standards of the institutional and/or national research committee and the 1964 Helsinki Declaration and its later amendments or comparable ethical standards. This study was approved by the Ethics Committee of Hirosaki University (reference numbers: 2021‐030 and 2021‐166‐3). Informed consent was obtained from all participants prior to their inclusion in the study.

## Data Availability

Data available on request due to privacy/ethical restrictions.
